# Computed tomography imaging characteristics of clear cell papillary renal cell carcinoma

**DOI:** 10.1590/S1677-5538.IBJU.2018.0716

**Published:** 2020-01-13

**Authors:** Taro Banno, Toshio Takagi, Tsunenori Kondo, Kazuhiko Yoshida, Junpei Iizuka, Masayoshi Okumi, Hideki Ishida, Satoru Morita, Yoji Nagashima, Kazunari Tanabe

**Affiliations:** 1 Tokyo Women's Medical University Department of Urology Department of Urology, Tokyo Women's Medical University, Tokyo, Japan; 2 Department of Urology, Tokyo Women's Medical University, Medical Center East, Tokyo, Japan; 3 Department of Diagnostic Imaging and Nuclear Medicine, Tokyo Women's Medical University, Tokyo, Japan; 4 Department of Surgical Pathology, Tokyo Women's Medical University, Tokyo, Japan

**Keywords:** Tomography, X-Ray Computed, Papillary renal cell carcinoma, sporadic [Supplementary Concept]

## Abstract

**Purpose:**

Clear cell papillary (CCP) renal cell carcinoma (RCC) is a new subtype of RCC that was formally recognized by the International Society of Urological Pathology Vancouver Classification of Renal Neoplasia in 2013. Subsequently, CCP RCC was added to the 2016 World Health Organization Classification of Tumors of the Urinary System and Male Genital Organs. In this study, we retrospectively investigated the computed tomography (CT) findings of pathologically diagnosed CCP RCC.

**Materials and Methods:**

This study included 12 patients pathologically diagnosed with CCP RCC at our institution between 2015 and 2017. We reviewed the patient's CT data and analyzed the characteristics.

**Results:**

Nine solid masses and 3 cystic masses with a mean tumor size of 22.7±9.2mm were included. Solid masses exhibited slight hyper-density on unenhanced CT with a mean value of 34±6 Hounsfield units (HU), good enhancement in the corticomedullary phase with a mean of 195±34HU, and washout in the nephrogenic phase with a mean of 133±29HU. The walls of cystic masses enhanced gradually during the corticomedullary and nephrogenic phases. Solid and cystic masses were preoperatively diagnosed as clear cell RCC and cystic RCC, respectively.

**Conclusions:**

The CT imaging characteristics of CCP RCCs could be categorized into either the solid or cystic type. These masses were diagnosed radiologically as clear cell RCC and cystic RCC, respectively.

## INTRODUCTION

Clear cell papillary (CCP) renal cell carcinoma (RCC) is a low-grade renal tumor that was first introduced into the International Society of Urological Pathology (ISUP) Vancouver Classification of Renal Neoplasia in 2013 (1). Subsequently, this entity was added to the 2016 edition of the World Health Organization (WHO) Classification of Tumors of the Urinary System and Male Genital Organs (2). CCP RCC is an indolent renal epithelial neoplasm characterized by a tubule-papillary structure of bland clear epithelial cells. The cancer cell nuclei exhibit a predominantly linear alignment away from the basement membrane, with low Fuhrman system nuclear grades of 1 or 2. CCP RCC lacks a sinusoid-like vasculature and thus differs from clear cell RCC. Moreover, the immunophenotypic factors of CCP RCC differ from those of clear cell RCC and papillary RCC (2). Although CCP RCC was initially thought to be limited to cases of end-stage kidney disease, several later reports described this malignancy in non-diseased kidneys (3, 4).

Generally, CCP RCC is diagnosed from surgical specimens following an initial classification as another type of RCC, such as acquired cystic disease of the kidney (ACDK)-associated RCC or cystic RCC, on preoperative imaging studies. As noted above, CCP RCC is a relatively new pathological subtype, and therefore few reports have described the radiological imaging findings of CCP RCC. In a recent study of 28 CCP RCCs, Wang et al. described two types of typical imaging findings, namely a solid mass with relatively low-level enhancement, as seen in papillary RCC, and heterogeneous regions of hyper-enhancement, as seen in clear cell RCC (5).

Although a few articles previously described the radiological findings of CCP RCC, the reported studies included relatively small numbers of patients. In this study, we retrospectively investigated the computed tomography (CT) findings of 12 patients pathologically diagnosed with CCP RCC at a single Japanese institution.

## MATERIALS AND METHODS

This retrospective study investigated the clinical, pathological and radiological findings of 12 patients who were pathologically diagnosed with CCP RCC between 2015 and 2017 at our institution.

CT images were obtained using a 64-row detector scanner (Aquilion 64; Toshiba Medical Systems, Otawara, Japan) with the following settings: pitch, 0.83; collimation, 0.5mm; reconstruction thickness/interval, 1.0mm/1.0mm and 120kVp with AutomA. Arterial phase images were obtained using a bolus-tracking contrast monitoring system after contrast material injection for 30 seconds. The total iodine dose was 600mg/kg body weight. Enhanced CT scans were performed at 40-, 90- and 300-seconds post-injection with fixed post-contrast timing, which corresponded to the corticomedullary, nephrogenic and excretory phases, respectively. Preoperative CT images were reviewed by at least two radiologists with more than 10 years of experience. For solid masses, the region of interest used to determine the Hounsfield unit (HU) measurement was placed over the area with the greatest attenuation. For cystic masses, the visual enhancement pattern and Bosniak classification were determined.

Pathological specimens were assessed using hematoxylin-eosin and immunohistochemical staining for cytokeratin (CK) 7, cluster of differentiation (CD) 10, α-methylacyl-CoA racemase (AMACR), transcription factor E3 (TFE3), cathepsin K, and carbonic anhydrase (CA) IX. At least two pathologists with 30 years of experience interpreted the results. The tissue slides were evaluated for growth patterns, architecture, and cellular characteristics. Tumors were staged according to the tumor-node-metastasis system (6) and graded according to the Fuhrman classification (7). The pathological types were determined according to the 2016 WHO Classification of Urogenital Tumors (2).

## RESULTS

### Clinical characteristics

The patient's characteristics are shown in Table-1. During the study period, 894 patients underwent radical or partial nephrectomy at our institution. Of these, 12 Japanese patients (1.3%) with CCP RCC (6 men, 6 women) were identified. These patients had a mean age at diagnosis of 56.3±10.9 years. Kidney tumors were detected incidentally in 9 of 12 patients, while two patients presented with flank pain and one presented with weight loss. Two of 12 patients had end-stage renal disease (ESRD) for which they underwent dialysis. Regarding the surgical procedure, 10 patients underwent partial nephrectomy; the two patients with ESRD underwent radical nephrectomy.

**Table 1:** Clinical characteristics.

Number of patients		12
Mean range (range) [y]		56.3 ± 10.9 (43-73)
**Gender (n, %)**		
	Male	6 (50)
	Female	6 (50)
**Clinical presentation (n, %)**		
	Incidental finding	9 (75)
	Flank pain	2 (16.7)
	Body weight loss	1 (8.3)
	End-stage renal disease (n, %)	2 (16.7)
**Surgical procedure (n, %)**		
	Radical nephrectomy	2 (16.7)
	Partial nephrectomy	10 (83.3)

### CT imaging

**Figure 1:**
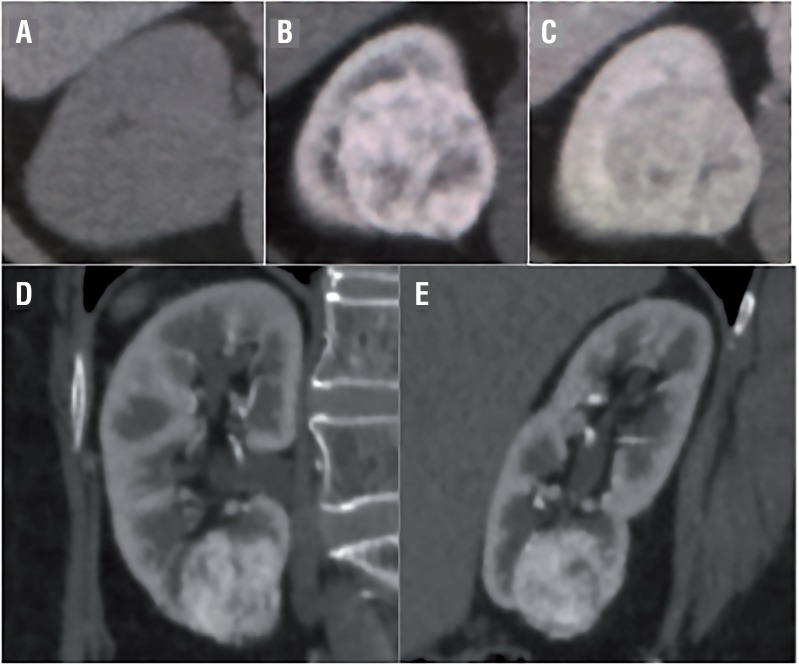
Solid pattern: Case 3.

**Figure 2:**
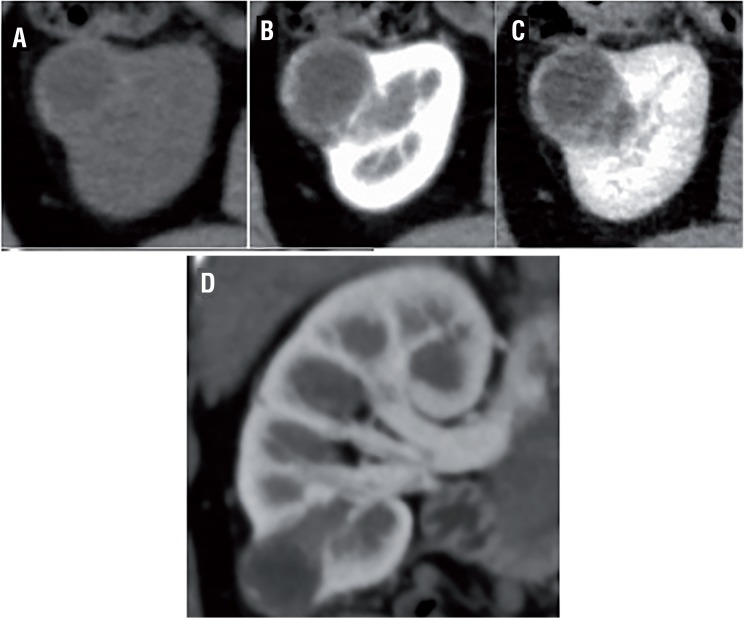
Cystic pattern: Case 11.

**Figure 3:**
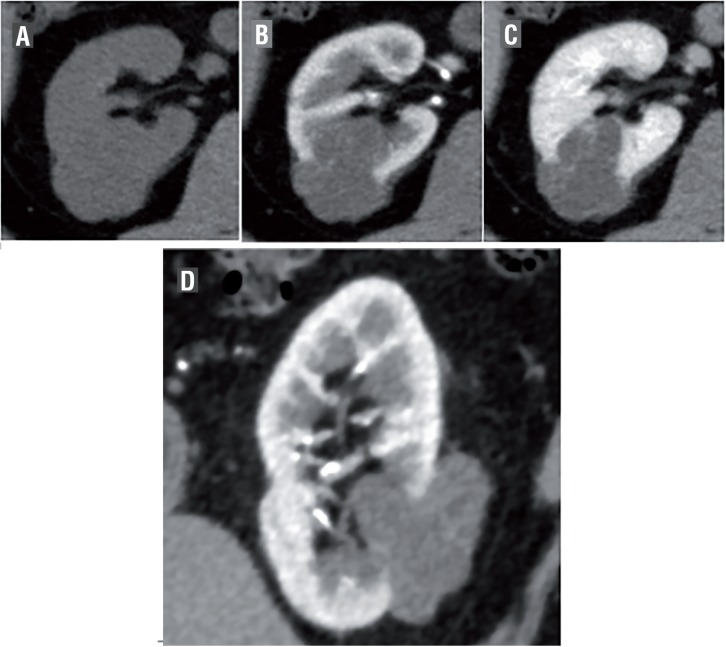
Cystic pattern: Case 12.

The CT findings are shown in Table-2 and Figures 1, 2 and 3. The mean tumor size was 22.7±9.2mm. Three tumors were exophytic (>50% projection beyond the renal parenchyma) and 9 were endophytic (<50% within the renal parenchyma). Nine and 3 cases involved solid and cystic masses, respectively. Regarding CT attenuation, all but one solid mass (exception: case 9) appeared as a mildly hyperdense area on unenhanced CT, with a mean value of 34±6HU. These areas exhibited good enhancement in the corticomedullary phase (CMP) with a mean of 185±45HU, and washout in the nephrogenic phase with a mean of 137±30HU as shown in Figure-1. Case 9 exhibited a solid mass (42HU) with ACDK, which was enhanced in both the corticomedullary phase (101HU) and nephrogenic phase (172HU). As shown in Figures 2 and 3, the walls and septum of small cystic lesions were enhanced gradually in the corticomedullary and nephrogenic phases.

a) Non-enhanced CT: Mild high density area (38HU).

b) Corticomedullary phase: Well enhanced (249HU).

c) Nephrogenic phase: Washed out (187HU).

d) Coronal slice imaging at the corticomedullary phase.

e) Sagittal slice imaging at the corticomedullary phase.

a) Non-enhanced CT: Mild density cyst.

b) Corticomedullary phase: Enhanced cyst wall.

c) Nephrogenic phase: The density of the cyst wall is almost the same with that of CMP.

d) Coronal slice imaging at the corticomedullary phase.

a) Non-enhanced CT: Mild density cyst.

b) Corticomedullary phase: Enhanced septum.

c) Nephrogenic phase: The density of the septum is almost the same with that of CMP.

d) Coronal slice imaging at the corticomedullary phase.

**Table 2:** CT imaging characteristics of clear cell papillary renal cell carcinoma.

Case	Age (year)	Sex	Tumor size (mm)	ESRD	CT pattern	Enhanced pattern (HU)			Preoperative diagnosis
						Plain	CMP	Nephrogenic
1	64	F	12		solid	37	216	150	Clear Cell RCC
2	52	M	21		solid	33	163	143	Clear Cell RCC
3	73	F	30		solid	38	249	187	Clear Cell RCC
4	45	F	28	yes	solid	25	173	94	Clear Cell RCC
5	54	M	16		solid	25	147	105	Clear Cell RCC
6	50	F	12		solid	35	219	118	Clear Cell RCC
7	62	F	20		solid	43	210	139	Clear Cell RCC
8	44	M	19		solid	33	185	129	Clear Cell RCC
9	68	F	28	yes	solid	42	101	172	ACDK associated RCC
10	71	M	13		cystic				Cystic RCC BosniakIV
11	43	M	32		cystic				Cystic RCC Bosniak III
12	49	M	41		cystic				Cystic RCC Bosniak III

**CMP=** corticomedullary phase

Eight of the 9 patients with solid masses received radiologic diagnoses of clear cell RCC. The ninth patient was diagnosed with ACDK-associated RCC. All patients with cystic masses received radiologic diagnoses of cystic RCC (2 Bosniak III cases and 1 Bosniak IV case).

### Histopathological findings

Table-3 summarizes the histopathological findings. The mean tumor size was 19.6±9.9mm. Eleven of 12 tumors were stage pT1a, while the twelfth was pT1b. Four and 8 tumors were classified as grade 1 and 2, respectively. Immunohistochemistry revealed CK7 positivity in all cases and CA-IX positivity in all but one case. However, CD10 positivity was observed in only 5 cases, while AMACR was positive in only one case. TFE3 and cathepsin K were negative in all cases.

**Table 3:** Histopathological findings.

Mean tumor size (range) [mm]		19.6 ± 9.9 (10-43)
**Pathological stage (n, %)**		
	T1a	11 (91.7)
	T1b	1 (8.3)
	≥T2	0 (0)
**Tumor grade (n, %)**		
	Grade1	4 (33.3)
	Grade2	8 (66.7)
	Grade3	0 (0)
	Grade4	0 (0)
CK7 positive (n, %)		12 (100)
CAIX positive (n, %)		11 (91.7)
CD10 positive (n, %)		5 (41.7)
AMACR positive (n, %)		1 (8.3)

## DISCUSSION

Our present study findings demonstrate that the radiological characteristics of CCP RCC can be categorized into two types: solid and cystic. Regarding the solid type, most CT scans showed that mildly hyperdense areas in the unenhanced phase were well enhanced in the corticomedullary phase and washed out in the nephrogenic phase, similar to clear cell RCC. All but one solid mass (exception: case 9 with ACDK) were radiologically diagnosed as clear cell RCC. Regarding cystic-type tumors, which were radiologically diagnosed as cystic RCC, the cystic walls of small lesions were gradually enhanced, and the HU values were nearly identical between the corticomedullary and nephrogenic phases. Our study demonstrated that all patients pathologically diagnosed with CCP RCC received radiological diagnoses of different subtypes. Therefore, the preoperative diagnosis of CCP RCC might be difficult.

To our knowledge, only two previous studies described the imaging characteristics of CCP RCC. Wang et al. reported the imaging characteristics of 28 CCP RCCs and demonstrated two types of typical findings, namely a solid mass with relatively low-level enhancement (similar to papillary RCC), and heterogeneous regions of hyper-enhancement (similar to clear cell RCC) (5). CCP RCC shares common pathological features with both clear cell RCC and papillary RCC, including cells with a clear cytoplasm and papillary architecture (8). These common pathological characteristics may be reflected in CT images as both clear cell and papillary RCC enhancement patterns. In contrast, we did not observe any enhancement patterns characteristic of papillary RCC, which might be due to the limited number of patients included in our study. Regarding the distinction of CCP RCC from other RCC subtypes via CT imaging, Mnatzakanian et al. reported the imaging characteristics of 18 CCP RCCs and compared these with the features of clear cell RCC and papillary RCC. They found that compared to papillary RCC, CCP RCC has a lower mean attenuation value on unenhanced CT (≤25HU), ill-defined margins, non-enhancing areas, and hyperintensity on T2-weighted magnetic resonance imaging. However, the authors noted no significant differences in imaging features between CCP RCC and clear cell RCC (9).

In the present study, a pathological analysis of CCP RCC revealed proliferating cells with clear cytoplasm that formed papillo-tubular, acinar, and cystic architectures, as described in the ISUP (1). Eleven and 1 case in the present study were staged as pT1a and pT1b, respectively, and 4 and 8 cases received Fuhrman grades of 1 and 2, respectively. No perirenal or lymphovascular invasion was noted. Taken together, these results further confirm the low malignant potential of CCP RCC (10). Immunohistochemistry can also be useful for distinguishing CCP RCC from other RCC subtypes. Although all RCC tumor cells are positive for PAX8, a marker of renal origin, the distinct immunophenotypic characteristics of CCP RCC include strongly diffuse CK7 and CA-IX expression. CA-IX staining reveals a cup-shaped and membranous distribution pattern with an absence of staining along the luminal borders of tumor cells. However, these cells are consistently negative for CD10, AMACR, cathepsin K and TFE3 (8, 11–15). In our study, CK7 positivity was observed in all cases, and all but one case exhibited CA-IX staining in the basolateral domains of tumor cells, which yielded giving a “cup-shaped” appearance.

In this study, CCP RCC was associated with ESRD in two patients who were submitted to hemodialysis, consistent with other studies (9, 16). As noted previously, CCP RCC was initially reported in patients with ESRD, but was later observed in non-disease settings. Aron et al. discussed the histopathologic features of these latter CPP RCCs and confirmed that this malignancy is a unique subtype of adult renal epithelial neoplasia in which the tumors are frequently small, have a low nuclear grade, and occur in same spectrum ranging from tumors occurring sporadically to those occurring in ESRD (4). In a previous investigation of the pathological characteristics of RCC in 408 patients receiving dialysis, we reported that 76%, 22% and 4% of the patients were diagnosed with cystic RCC, papillary RCC and other types of RCC, respectively (17). Given the relatively recent distinction of CCP RCC, the low-grade nature of this malignancy and the recent inclusion of this tumor in the WHO Classification of Tumors of the Urinary System and Male Genital Organs (2), the pathological distribution of CCP RCC in dialysis patients with RCC may differ following the application of new criteria.

CCP RCC has an excellent prognosis, which may reflect the low malignant potential (10). Massari et al. reviewed 24 publications that reported follow-up data of 362 patients with CCP RCCs/renal adenomatoid tumors and examined the prognoses and outcomes. Notably, no cases of local recurrence, lymph node or distant metastasis and disease-related death were reported during a mean follow-up duration of 38 months (18). Similarly, we did not observe any recurrences, metastases, or disease-related mortality during a mean follow-up duration of 14±6 months. However, the imaging findings do not always reflect the low malignant potential of CCP RCC according to the pathological findings. According to Wang et al. CCP RCCs exhibited a mean growth of 0.6cm (±0.4cm) during a mean follow-up period of 811 days (5), compatible with the observed growth of other low-stage RCCs (19). Moreover, the imaging-based preoperative diagnosis of CCP RCC may be difficult, as discussed previously. Generally, solid-type CCP RCC is diagnosed radiologically as a clear cell or papillary RCC, according to the enhancement pattern. These growth or radiographic patterns may indicate the need for surgical intervention. However, pre-operative CT-guided biopsy is a good diagnostic option for all RCCs except the cystic type, which may yield an insufficient specimen amount due to cystic fluid (20, 21). Given the low malignant potential of CCP RCC, careful follow-up or minimally invasive treatment (e.g., cryotherapy and radiofrequency ablation) should be considered.

This study had some limitations, including its retrospective nature. Moreover, the generalizability of the results may be affected by the single tertiary care institution setting. Still, CCP RCC was only recently defined, and therefore few reports have described this pathological subtype, particularly with regard to radiological findings. Our results therefore provide a useful reference for the diagnostic imaging of CCP RCC, despite the small sample size.

In conclusion, the CT imaging characteristics of CCP RCCs can be divided into solid and cystic types. Although CCP RCC is considered to have a low malignant potential, it is difficult to distinguish this subtype from other subtypes of RCC based on CT imaging features alone.

## ABBREVIATIONS

CCP: clear cell papillary
RCC: renal cell carcinoma
ISUP: International Society of Urological Pathology
WHO: World Health Organization
CT: computed tomography
HU: Hounsfield unit
CK: cytokeratin
CD: cluster of differentiation
AMACR: á-methylacyl-CoA racemase
TFE3: transcription factor E3
CA: carbonic anhydrase
ESRD: end-stage renal disease
CMP: corticomedullary phase
ACDK: acquired cystic disease of the kidney
